# The Premise of the Paradox: Examining the Evidence That Motivated GIPR Agonist and Antagonist Drug Development Programs

**DOI:** 10.3390/jcm14113812

**Published:** 2025-05-29

**Authors:** Jonathan D. Douros, Stephanie A. Mowery, Patrick J. Knerr

**Affiliations:** Indiana Biosciences Research Institute, Indianapolis, IN 46202, USApknerr@indianabiosciences.org (P.J.K.)

**Keywords:** GIP, GIPR, GLP-1, GLP-1R, obesity, pharmacology, incretin, MariTide, tirzepatide

## Abstract

Emerging clinical data support the paradoxical notion that glucose-dependent insulinotropic polypeptide (GIP) receptor (GIPR) agonism and antagonism can provide additive weight loss when combined with a glucagon-like peptide 1 (GLP-1) receptor (GLP-1R) agonist. In this review, we examine data that motivated the initiation of these seemingly contradictory drug discovery programs. We focus on the physiologic role of GIP in humans, human genetics evidence, rodent genetic models, and preclinical rodent and non-human primate pharmacology studies. Furthermore, we highlight where early preclinical findings translated into relevant clinical efficacy in the development of tirzepatide and maridebart cafraglutide (MariTide).

## 1. Introduction

Obesity poses a significant burden to individual and public health [1]. GLP-1R agonists have proven highly effective in combating the disease [2,3], however, there is persistent unmet patient need due to efficacy and tolerability limitations as well as supply constraints. This suggests a need for new mechanisms of action (MoA) and drug modalities. The GIPR has emerged as a therapeutic target over the last 15 years, though its development has lagged considerably behind that of its sister incretin receptor GLP-1R. Remarkably, the fundamental question of whether to agonize or antagonize GIPR to treat obesity remains unanswered given the clinical success of both the dual GLP-1R/GIPR co-agonist tirzepatide [4] and the GIPR antagonist antibody/GLP-1R peptide agonist conjugate maridebart cafraglutide (MariTide, formerly AMG-133) [5]. The clinical data for these programs has been discussed elsewhere [6]. In this review, we will examine data that motivated the initiation of these seemingly contradictory drug discovery programs.

## 2. In Favor of Antagonism

GIP is an intestinally derived, insulinotropic hormone commonly referred to as an incretin (for a comprehensive review see [7]). While the physiologic GIP action to potentiate insulin secretion and improve glycemia in healthy volunteers suggests pharmacologic agonism as a means to treat metabolic disease, this argument is not wholly commended by studies of patients with obesity and/or T2D. Exogenous GIP-potentiated insulin secretion is nearly ablated in patients with T2D [8,9]. Furthermore, GIP co-infusion does not provide additive glycemic control beyond that of GLP-1 infusion in patients with T2D, due in part to its action as a glucagon secretagogue [10]. This suggests GIPR agonists would be ineffectual in patients with T2D and potentially counteract the actions of GLP-1R agonists. Furthermore, native GIP infusion alone fails to reduce food intake in patients with obesity nor does it drive additional reduction in energy intake when paired with the GLP-1R agonist liraglutide [11,12]. In fact, GIP infusion actually reverses the hypophagic effect of native GLP-1 infusion [11], although this may be attributable to an anti-nauseating effect of GIPR agonism that has recently been described [13,14,15]. While these data do not speak directly to the potential efficacy of GIPR antagonists as weight reducing pharmacologic agents, they do provide a deterrent from developing GIPR agonists for T2D and obesity.

The physiologic and pathophysiologic role of GIP as an adipogenic factor in humans provides an initial rationale for the development of GIPR antagonists to treat obesity. Endogenous GIP is secreted most potently in response to high lipid meals [16], while GIP infusion enhances adipose tissue blood flow in lean patients [17] and lipid deposition (e.g., reduced plasma non-essential fatty acids (NEFA), increased adipose triglycerides (TG)) in patients with obesity and type 2 diabetes (T2D) [18]. These functions are reduced to control levels by the GIPR antagonist GIP_(3-30)_NH_2_ [17]. Thus, GIP serves a physiologic function to respond to lipid ingestion and promote lipid storage. Prandial GIP levels have deleterious, positive correlations with waist-to-hip ratio and visceral fat in men, but also desirable correlations with subcutaneous fat and cholesterol profiles [19]; it should be noted that significant divergence is present between male and female volunteers. Furthermore, prandial GIP levels are elevated in patients with T2D and obesity [20] and are positively correlated with obesity [21]. These patient data collectively paint a picture of GIP as an obesogenic factor in the context of overfeeding, which responds to lipid intake by stimulating lipid deposition that is at least partially mediated by the insulin stimulatory effect of the hormone.

Preclinical evidence supporting GIPR antagonism emerged from studies using mice with a global, germline GIPR knockout (KO) [22,23]. These animals have no body-weight phenotype on a chow diet, but they are protected from diet-induced obesity (DIO) when placed on a high fat diet (HFD). This phenotype was associated with a shift toward lipid catabolism, a reduction in adiposity [23,24] and, to a lesser extent, lean mass [25], with no change in food intake. These studies comport with the human data to suggest GIP acts as an obesogenic factor in the context of overnutrition. Modifications to lipid catabolism and deposition in global GIPR KO mice implicates an adipocyte-centric mechanism. However, inducible, adipocyte-specific GIPR deletion only partially recapitulates the global KO phenotype [26], while re-expression of the GIPR in adipocytes exacerbates HFD-induced weight gain but not adiposity [25]. Specific deletion of GIPR in the brown adipose tissue failed to reproduce any of the global GIPR KO phenotype [27] while β-cell specific GIPR deletion results in reduced adiposity in mice, but does not provide protection from DIO [28]. Normalizing deficient prandial insulin in β-cell GIPR KO mice restored adiposity to control levels, further implicating the insulin stimulatory effect of GIP in this phenomenon as seen in the human data. In general, peripheral deletion of the GIPR has recaptured aspects but not the totality of the global GIPR KO phenotype. On the other hand, deletion of broad central nervous system (CNS) [29,30] and GABAergic neuron specific [31] GIPR populations produces a DIO-protective phenotype along with reduced fat mass. Yet, these two mouse lines do exhibit decreased food intake, unlike the global GIPR KO line, which has not been fully explored. Collectively, these rodent genetics models suggest an incompletely described MoA by which adipocyte, β-cell, and CNS GIPR populations differentially regulate lipid accumulation, food intake, and ultimately body weight. While the precise mechanisms are not fully elucidated, the data do provide a clear rationale for GIPR antagonism to provide metabolic benefit by reducing overall body weight with a specific reduction in adipose mass. A key countervailing, but frequently overlooked, issue with this argument is that whole body GLP-1R KO mice demonstrate much the same phenotype as the GIPR KO animals [32]. This calls into question the translational relevance of incretin receptor knockout mouse physiology to human incretin antagonizing pharmacology.

Compelling preclinical pharmacologic data from both academic and industry groups demonstrate that GIPR antagonists induce substantial weight loss when administered in combination with a GLP-1R agonist. Killion et al. show that a potent GIPR antagonizing antibody nearly abolishes HFD induced weight gain in mice and decreases both fat mass and fasting insulin [33], phenocopying the global GIPR KO mice. They also report only minor reductions in lean mass compared to controls, suggesting positive differentiation from other weight loss therapy MoAs. While the GIPR antagonist alone provides little weight loss in DIO mice, its combination with the GLP-1R agonist liraglutide nearly doubles the weight loss seen with liraglutide alone. This corresponds with additional fat mass reduction and improved oral glucose control, but not food intake reduction, hinting at a novel MoA as compared to GIPR agonists [29,31,34]. The authors further demonstrate that the GIPR antagonist can induce additional weight loss beyond the maximal effect of liraglutide and, critically, that the effect translates to non-human primates (NHPs). Similar outcomes have been reported by others using a different antibody antagonist [35] or a GIPR antagonizing peptide [36]. While one preclinical report shows no additional weight loss when combining GIPR antagonism with GLP-1R agonism [34]; the tool compound was later demonstrated to have a pharmacokinetic liability [36]. Recent data demonstrates that GLP-1R agonizing peptide/GIPR antagonizing antibody conjugates similar to MariTide require CNS GIPR populations to drive weight loss. This dovetails with our own recently published work demonstrating that, unlike GIPR agonists, GABAergic neuronal GIPR populations are not required for the weight lowering effects of a peptide GIPR antagonist dosed in combination with a GLP-1R agonist [37]. The collective data strongly suggest that GIPR antagonism primarily acts on GABAergic neurons in the CNS to potentiate the effects of GLP-1R agonists which act on glutamatergic neurons to reduce food intake [37].

While the available clinical data for GIPR-modulating drugs have been recently compiled [6], a variety of factors impacting the clinical development of GIPR antagonists for the treatment of obesity deserve consideration, including the choice of modality for the active pharmaceutical ingredient (API), dose size, and potential inhibition of beneficial physiological effects of GIP. There is a GIPR small molecule antagonist (Pfizer, PF-07976016) in phase 2 clinical trials (NCT06717425) but little information is currently available regarding its structure, in vitro profile, or in vivo efficacy. Our own programs generated optimized, peptide-based GIPR antagonists suitable for once-daily administration but required enormous doses to achieve in vivo efficacy [36]. Attempts to extend the dosing interval using fatty diacid protraction moieties made our compounds insufficiently potent. These liabilities appear to have been overcome by the GIPR antagonist peptide AT-7687 under development by Antag Therapeutics, though no chemical structure has yet been disclosed [38]. On the other hand, Veniant et al. report that MariTide, a GIPR antagonizing antibody conjugated on each heavy chain to a GLP-1R agonizing peptide, makes significant gains on peptide-based antagonists due to its high potency at both receptors and suitability for once-monthly administration in humans [5]. Three doses of MariTide drove ~15% weight loss in patients with obesity which was maintained for nearly 60 days following the last dose. It is not clear whether the maintenance of weight loss after discontinuation of dosing is due simply to the impressive pharmacokinetics of MariTide or some unknown MoA of the pharmacology. Despite the positive results, MariTide requires a substantial injection volume and large doses (420 mg at 70 mg/mL, total 6 mL administration) to achieve efficacy on par with the GLP-1R agonist semaglutide (up to 2.4 mg/0.5 mL injection). Thus, from a patient use and manufacturing perspective, the biochemical optimization of GIPR antagonists may require further refinement. Additionally, despite the profound weight loss, patients experience a numeric elevation of free-fatty acids at the highest dose (420 mg) which declines to baseline during the washout phase [5]. This feature of the GIPR antagonist is incongruous with the physiologic role of GIP and differentiates from that of dual incretin agonists like tirzepatide which lowers triglycerides and free fatty acids in the clinical and preclinical setting.

## 3. Human Genetics Data in Favor of Both Agonism and Antagonism

There is increasing reliance on human genetics data to help identify new drug targets; but in the case of GIPR, these data provide a mixed picture. Missense GIPR variants (Arg190Gln, Glu288Gly, and Glu354Gln) have been separately reported to associate with increased body mass index (BMI) [39,40] and prandial glucose dysregulation [41] but also decreased adiposity. Others report that enhanced GIPR signaling (proxied by GIPR variants with reduced T2D risk) is associated with reduced BMI and triglyceride levels [42]; this analysis assumes that increased GIPR action reduces T2D risk which is not fully established. These conflicting findings highlight the difficulty in neatly (transcribing and) translating genetic data into pharmacologic interventions. As discussed below, factors including which population of receptors, the receptor pharmacology, and disease state to target all require consideration in determining whether agonism or antagonism of the GIPR is appropriate.

## 4. In Favor of Agonism

While GIP serves as the primary incretin in healthy humans [43], its use as an anti-diabetic agent was initially discouraged by the finding that the insulinotropic action of GIP infusion is severely diminished in patients with T2D [8]. However, this phenomenon does not necessarily translate to chronic treatment with long-acting GIPR agonists as we shall see. A key initial observation was that GIPR desensitization in the T2D model streptozotocin-treated mouse was ameliorated after glucose normalization using sulfonylureas. This provided a rationale for combining GLP-1R agonists, which effectively lower blood glucose in T2D, with GIPR agonists. Under this paradigm, GIPR agonism would presumably gain efficacy throughout treatment as glycemia normalized.

Indeed, seminal preclinical pharmacology by Finan et al. demonstrated that long-acting GIPR monoagonists, delivered alone or in combination with GLP-1R agonists, effectively lowered body weight and food intake in diet-induced obese (DIO) mice while also improving glucose control [44]. In this same work, the authors disclosed rationally-designed, long-acting, unimolecular dual GLP-1R/GIPR agonists for the first time. These molecules elicited additive effects to reduce weight and improve glucose control beyond the loose combination of pharmacokinetically and potency-matched GLP-1R and GIPR mono-agonists. Similarly, rationally designed unimolecular triagonists for the GLP-1R, GIPR, and glucagon receptor (GCGR) developed by Knerr et al. and Finan et al. have been shown to produce additive weight loss over GLP- 1R/GCGR dual agonists [44,45]. This likely also applies to clinical triagonists including retatrutide [46], SAR441255 [47], and NN1706 [48] but has not been actively demonstrated. These pieces of data collectively indicate that GIPR agonism, either alone or in combination with other weight-lowering agents, can drive significant metabolic benefits. This finding appears to translate to humans given that the GIPR mono-agonist LY3537021 has been reported to reduce body weight in healthy volunteers and patients with T2D [15].

Mechanistic work has focused on the role of GIPR agonists to reduce food intake by acting in the central nervous system (CNS). GIPR agonism acts in the CNS [29], specifically on VGAT+ GABAergic neurons [31], to reduce food intake and body weight. Key data indicate that knocking out GIPR in GABAergic (VGAT+) neurons ablated the ability of GIPR mono-agonists to drive weight loss on their own and for dual GLP-1R/GIPR agonists to produce additive weight loss beyond GLP-1R agonists alone [31]. In contrast, knocking out GIPR in glutamatergic (VGLUT2+) neurons had a negligible effect on body weight reductions by these agonists [49]. This is in contrast to GLP-1R agonists, which require glutamatergic but not GABAergic neurons to drive food intake reduction and weight loss [50].

Beyond the CNS mechanisms that reduce food intake, Samms et al. [51,52] and Regmi et al. [53] have demonstrated that GIPR agonism via tirzepatide can improve insulin sensitivity in the adipocytes of DIO rodents. A key feature of the studies from Regmi et al. is the differences in the effect of GIPR agonism during the fed and fasted states. Physiologic GIP is secreted upon feeding, where it stimulates insulin secretion and promotes glucose and lipid uptake in the adipocyte, as previously discussed. This drove the conclusion that GIP is adipogenic and that the receptor should be antagonized to treat obesity. However, the paradigm is notably different from a long-acting agonist, where GIPR stimulation is chronically high in both the fed and the fasted state. It is during the fasted state when insulin levels are low that GIPR agonism actually promotes lipolysis and lipid efflux [53]. This effect, combined with the negative energy balance driven by low food intake of a dual GLP-1R/GIPR agonist, promotes a shift to lipid catabolism, as noted repeatedly in rodent studies. This phenomenon appears to hold true in the clinical setting as humans undergo a significant shift toward lipid catabolism during tirzepatide treatment [54].

Finally, GIPR agonism is increasingly appreciated to reduce the aversive effects typically seen with GLP-1R agonists [55]. This phenomenon is most clearly seen in shrews which exhibit reduced food intake and improved glucose control without emesis when treated with a GIPR mono-agonist [14]. Critically, the emetic effects of GLP-1R agonism are blunted by GIPR agonists without a reduction in glucose lowering efficacy in the shrew model. A similar outcome has been seen in clinical studies of a GIPR mono-agonist LY3537021 paired with liraglutide [15].

## 5. Synthesis

Three primary hypotheses have emerged to explain these paradoxical findings that both GIPR agonism and antagonism can be coupled with GLP-1R agonism to drive additional body-weight loss beyond that of GLP-1R agonism alone.

The first is that the GIPR agonism has no effect on its own and that the improvements in weight loss for tirzepatide over the GLP-1R monoagonist semaglutide are due to a Gs-biased GLP-1R agonism profile [56,57]. Gs-biased GLP-1R agonists, characterized by partial Gs recruitment/activation but disproportionately reduced β-arrestin recruitment, have been demonstrated to confer superior maximal body weight reduction in preclinical models when compared to balanced agonists such as exendin-4 and semaglutide [58,59,60,61]. However, this does not necessarily exclude GIPR agonism as a mechanism for improving the efficacy of tirzepatide over semaglutide. Our work demonstrates that tirzepatide indeed exhibits GIPR agonism in the islets of healthy human donors [62]; however, this does not account for the reduction in GIPR sensitivity in patients with T2D or the potential differences in GIPR agonism between islet cells and food-intake regulating neurons. Nevertheless, the food intake and weight reducing effects of GIPR mono-agonism in humans suggests that GIPR agonism of tirzepatide is indeed playing a role in the drug’s clinical efficacy. Two things can be true simultaneously; both biased GLP-1R agonism and GIPR agonism can contribute to the efficacy of tirzeaptide.

The second explanation is that chronic GIPR agonism induces functional antagonism [33,63]. G-protein coupled receptors (GPCRs) like the GIPR are widely known to undergo ligand-mediated internalization, which serves to functionally desensitize signaling by the receptor [64,65]. Theoretically, chronic stimulation can induce receptor desensitization resulting in functional antagonism. Killion et al. tested this concept by treating primary adipocytes with an acylated GIPR agonist to achieve prolonged GIPR activation, washed out the agonist, then restimulated the GIPR [63]. They demonstrate that prolonged (24 h) agonism with high dose (1 μM) GIP does indeed elicit near complete functional desensitization of the receptor to secondary stimulation as measured by fatty-acid uptake in murine and human adipocytes. In these studies adipocytes are still able to respond to GIP restimulation after shorter pre-treatment time courses (1–4 h) or lower pretreatment doses (10–100 nM). It is unclear how the duration and dosing used in this experimental system reflects the in vivo setting, though it would be of interest to benchmark the degree of desensitization/functional antagonism achieved in these studies to the GIPR antagonizing antibodies advancing through the clinic [5] or peptide antagonists reported in the literature [36]. It is challenging to reconcile the functional antagonism hypothesis with the observation that an agonist delivered at much lower doses achieves a comparable degree of weight loss to an optimized antagonist. This suggests that the agonist would need to possess exceptionally high antagonistic properties at relatively low exposures in vivo.

**Figure 1:**
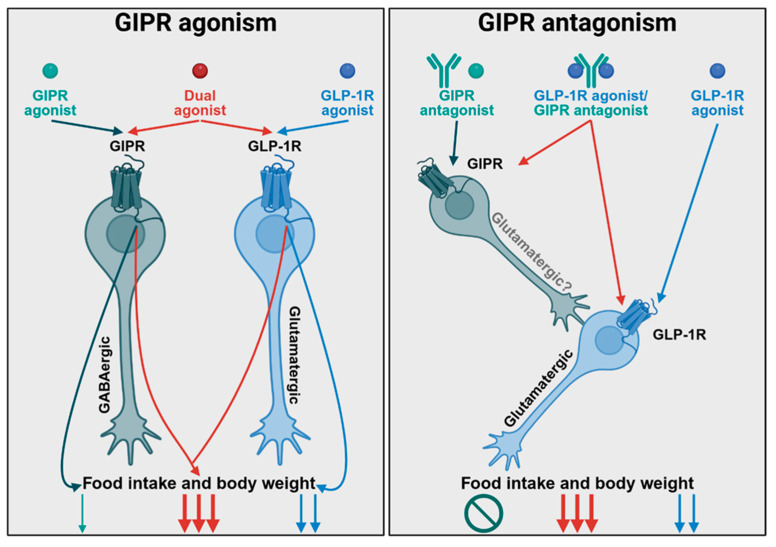
A proposed mechanism for the additive effects of GIPR agonists or antagonists with GLP-1R agonists on food intake and body weight. GIPR agonists can drive significant weight loss by acting on GABAergic neurons either alone or in combination with GLP-1R agonists, which act on glutamatergic neurons. Conversely, GIPR antagonists do not act through GABAergic neurons and require combination with GLP-1R agonism for substantial efficacy, suggesting they likely alleviate a tonic inhibition on the GLP-1R signaling system in the CNS. Downward arrows indicate a decrease in food intake; the number of arrows indicates the relative decrease. A crossed circle indicates no effect. Light green indicates the effect of GIPR agonists alone, light blue indicates the effect of GLP-1R agonists alone, and red indicates the effects of combined GLP-1R/GIPR agonism.

Third, there is compelling evidence that GIPR agonism and antagonism act via different neuronal mechanisms to drive weight loss. While preclinical evidence demonstrates both peptide and antibody GIPR antagonists drive modest weight loss in rodents on their own (<5% in DIO mice) [33,36], they appear to require combination with GLP-1R agonism to drive greater efficacy (>20% weight loss in DIO mice). The effects of the antagonists require intact action at CNS GIPR neurons [30], but not GABAergic populations [37], making it likely that these effects are mediated by glutamatergic GIPR+ neurons. Crucially, the neuronal transcriptomic changes induced by peptide GIPR antagonism are significantly different from those of GIPR agonism but closely mirror that of GLP-1R agonism [37]. Conversely, GIPR agonism alone can drive significant weight loss by acting on CNS GABAergic but not glutamatergic neurons [31,34,37,49]. These effects add to, and even synergize with, that of GLP-1R agonism. These data collectively suggest a unified theory in which that GIPR agonism drives efficacy both on its own and in combination with GLP-1R agonism by acting on receptors in GABAergic neurons, while GIPR antagonism in glutamatergic neurons primarily serves to alleviate a brake on GLP-1R action (Figure 1).

## 6. Conclusions

**Figure 2:**
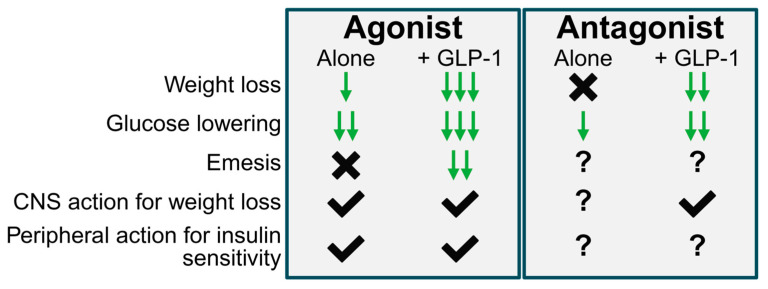
A summary of the actions of GIPR agonists and antagonists with and without GLP-1R agonists in preclinical studies on key outcomes for obesity therapeutics. Downward arrows indicate a reduction in the parameter; 1 arrow indicates a smaller reduction relative to 2. An X indicates no effect of the drug, a check mark indicates an action of the drug, a question mark indicates an unknown quality of the drug.

While the advent of GLP-1R agonists have indisputably revolutionized the pharmaceutical intervention of obesity, further improvements in efficacy and tolerability are necessary to maximize the long-term health outcomes of patients. Innovative drug discovery programs that enhance GLP-1R agonism with distinct but complimentary mechanisms of action, such as modulation of GIPR, are providing a new generation of such treatments. But the appropriate direction of that modulation has remained a stubborn source of controversy, as both agonists and antagonists of GIPR have advanced to and through clinical development with similar successes to treat the same condition (Figure 2). The physiologic role of GIP to regulate prandial glucose and lipid metabolism coupled with numerous preclinical studies commend GIPR antagonism as the appropriate mechanism to treat obesity. Indeed, GIPR antagonists of varying chemical modalities have demonstrated compelling preclinical and clinical effects to potentiate GLP-1R-induced weight loss. And yet, undeterred, dual agonism of GLP-1R and GIPR has also progressed and, in fact, achieved regulatory approval first for both T2D and obesity indications in the form of unimolecular co-agonist tirzepatide. Emerging evidence has identified additional pharmacological benefits of GIPR agonism beyond its well-established physiological incretin effect, such as reduction of food intake directly via CNS signaling, promotion of lipolysis in the fasted state, and improvement of GLP-1R agonist tolerability. All these activities may contribute to the superior weight-lowering efficacy of dual GLP-1R and GIPR agonism compared to GLP-1R agonism alone. A unified mechanisms for these opposing modulations of GIPR is emerging in which antagonists act on non-GABAergic neurons to potentiate GLP-1R induced hypophagia while agonists act on GABAergic neurons to reduce food intake on their own and in combination with GLP-1R agonists. Considering the persistently growing momentum surrounding incretin pharmacology, further insights into this unresolved paradox of GIPR agonism versus antagonism are undoubtedly on the horizon.
